# The impact of real-time computerised video analysis and feedback on hand hygiene practice and technique on a surgical ward

**DOI:** 10.1186/1753-6561-5-S6-O52

**Published:** 2011-06-29

**Authors:** A Ghosh, G Lacey, C Gush, S Barnes

**Affiliations:** 1 Computer Science and Statistics, Trinity College Dublin, Dublin 2, Ireland; 2 Department of Health, London, UK; 3 Mid Essex Hospital Services, Chelmsford, UK

## Introduction / objectives

The fast hand movement and occlusion of observers' views make it difficult to audit hand hygiene technique. We investigate the effect of computerised video observation and real-time feedback on the hand hygiene technique.

## Methods

Hand wash monitors (SureWash, Ireland) were placed above clinical sinks in a 28 bed surgical ward. The clinical trial consisted four phases: Phase 1 (1 week) determined the baseline practice without any feedback. Phase 2 (4 weeks) provided real-time feedback. In Phase 3 (5 weeks) a printed report was also presented at the weekly staff meeting. In Phase 4 (1 week) the feedback was turned off. The feedback was shown on a computer screen of each unit. SureWash deemed a hand washing complete if it followed every step of the CleanYourHands protocol.

## Results

The number of hand wash events (HWE) for each day was divided by the product of the number of patients and staff. The daily averages of HWE were 0.14±0.01, 0.36±0.02, 0.35± 0.02, 0.18±0.2 for phases 1, 2, 3 and 4 respectively. The increase between phase 1 and 2 was 156% (p<10^-7^) and the fall in phase 3 from 4 was 48% (p<10^-4^). The daily average number of completed HWE was 0.02±0.004, 0.17±0.01, 0.16±0.18, 0.02±0.005 in phases 1, 2, 3 and 4 respectively. The increase between phase 1 and 2 was 703% (p<10^-9^) and the fall in phase 3 from 4 was 48% (p<10^-3^). The total completion rates were: 15.8% (38/240), 49.1% (719/1464). 44.4% (724/1630) and 13.3% (24/180) in each phase respectively.

## Conclusion

Real-time computerised feedback on proper technique resulted in a significant increase in the number of HWE (+156%) and in the adherence (+703%) to the CleanYourHands protocol. Feedback acted as a reminder of technique and provided instruction on "difficult" poses.

## Disclosure of interest

A. Ghosh: None declared, G. Lacey Shareholder of GLANTA Ltd, C. Gush: None declared, S. Barnes: None declared.

